# Early and Innovative Rehabilitation in Warkany Syndrome 2 Associated with Agenesis of the Corpus Callosum: A Case Report

**DOI:** 10.3390/children9050722

**Published:** 2022-05-14

**Authors:** Carmela Settimo, Lilla Bonanno, Maria Tresoldi, Rosalia Muratore, Francesca Cucinotta, Emanuela Tripodi, Adriana Piccolo, Smeralda Anchesi, Caterina Impallomeni

**Affiliations:** IRCCS Centro Neurolesi Bonino Pulejo, 98124 Messina, Italy; carmela.settimo@irccsme.it (C.S.); lilla.bonanno@irccsme.it (L.B.); maria.tresoldi@irccsme.it (M.T.); rosalia.muratore@irccsme.it (R.M.); francesca.cucinotta@irccsme.it (F.C.); emanuela.tripodi@irccsme.it (E.T.); adriana.piccolo@irccsme.it (A.P.); smeralda.anchesi@irccsme.it (S.A.)

**Keywords:** Warkany syndrome 2, agenesis of the corpus callosum, virtual reality, rehabilitation, neuroplasticity, case report

## Abstract

Trisomy 8 mosaicism syndrome (T8MS) or “Warkany’s syndrome 2” is a rare chromosomal disorder characterized by three copies of chromosome 8 in some cells of the body. T8MS incidence in the world population is about 1/25,000–50,000 live births with a 5:1 ratio between males and females. Since chromosomal mosaicism is often present in this syndrome, affected subjects present a phenotype varying from mild dysmorphism to severe structural anomalies. Malformations, including corpus callosum agenesis and renal abnormalities, have been described by many studies. We present a case in a girl 36 months in age, born to assisted fertilization (FIVET) and prenatal diagnosis by amniocentesis. In a fetus in the 22 week of gestation, she presented trisomy 8 mosaicism with ventriculomegaly, agenesis of the corpus callosum and a sequence of polymalformations. Through the early identification of symptoms that gradually occurred during development, the girl was submitted, early, to innovative complex instrumental using virtual reality (VR) rehabilitation. This study involves continuous monitoring and early management of symptoms, with the aim of improving the neurobehavioral outcomes of children with this rare disease by inducing structural neuroplastic responses and significantly reducing the impact that this disorder has on the development of children born without corpus callosum.

## 1. Introduction

Trisomy 8 mosaicism syndrome (T8MS) or “Warkany’s syndrome 2” characterized by three copies of chromosome 8 in some cells of the body. This is a rare condition with an estimated frequency of approximately 1:25,000 to 1:50,000 children born alive with a 5:1 ratio between males and females. [1] Complete trisomy 8 is usually lethal, and 0.8% of first trimester miscarriages result form it, but fetuses with mosaic trisomy 8 are compatible with viability [2,3].

Since chromosomal mosaicism is often present in this syndrome, the affected subjects have a phenotype ranging from mild dysmorphism to severe structural abnormalities [4,5,6]. Although prediction of the phenotype is difficult [7,8], trisomy 8 mosaicism is commonly expressed as an increase in birth weight, accelerated somatic development, psychomotor retardation, mental retardation, prominent forehead, strabismus, upturned nose with broad bridge, low dysmorphic ears, inverted lower lip, slender trunk, osteoarticular anomalies, clinodactyly and deep skin furrows on the soles of the feet highly characteristic in trisomy 8 mosaicism [8,9,10,11]. 

Structural malformations, including corpus callosum agenesis (ACC) and renal abnormalities, have been described by many studies in subjects with trisomy 8 mosaicism [6,12,13]. The corpus callosum (CC), the main interhemispheric commissure, transfers cognitive, sensory and motor information between the two cerebral hemispheres [14]. It is believed that in 30–50% of cases the ACC is of genetic origin, associated with numerous chromosomal loci [15,16]. Numerous chromosomal loci have been associated with ACC [17], including trisomy 8 mosaicism [18,19]. Injuries in its context lead to mental disorders, with incoordination of ideation, character changes and especially alterations in the execution of movements (motor apraxia), cognitive and social difficulties linked to the failure to transfer such information from one hemisphere to another [14,19]. However, as is frequently reported in the literature, patients with trisomy 8 mosaicism associated with agenesis of the corpus callosum and the upper dislocation of the third ventricle, the degree of enlargement of the ventricles and a significant dilation of the interhemispheric fissure are more associated with clinical pictures with worse prognostic value [5]. Infants with corpus callosum agenesis may initially show no symptoms, particularly in the absence of other associated conditions. Growing up, they may experience common symptoms, such as seizures; psychomotor delays (poor muscle tone and coordination); cognitive deficits such as learning difficulties, problems with abstract thinking and problem solving and attention deficit disorder (ADD); eating disorders and swallowing difficulties; impaired language skills; impaired vision and hearing; insomnia or other sleep problems; and psychosocial difficulties such as obsessive behaviors and social immaturity.

Currently, it is not possible to restore the corpus callosum to normal, but agenesis of the corpus callosum, to manage any complications that may arise, has usually been treated with a multidisciplinary treatment that includes the following:

Pharmacological treatment to control epileptic seizures; 

Speech therapy to implement language skills;

Neuromotor therapy to counteract hypotonia and improve coordination;

Psychomotor treatment and occupational therapy to reduce cognitive problems and develop personal autonomy [20].

In recent decades, numerous studies in animal and human models have demonstrated that rehabilitation approaches based on environmental enrichment exert profound effects on the CNS. Habilitative treatment is more likely to be effective if it is applied early in development and mediated by multisensory stimulation, stimulating increased plasticity in the brain. These results are very promising for the field of neurorehabilitation [21,22]. In a 2011 study [23] highlighted the mechanisms of anatomical and functional reorganization in subjects with isolated and complete agenesis of the corpus callosum and normal cognitive abilities. In this context, it is possible to use Virtual Reality (VR) technology, a new tool with a possible added value compared to traditional neurorehabilitation. However, despite the fact that in the last decades knowledge in the field of child development is supported by extensive scientific literature, there are few studies on the role of VR training in the cognitive rehabilitation (CR) of patients with T8MS and agenesis of the corpus callosum.

This study aims to evaluate the impact that a conventional habilitative treatment associated with an instrumental treatment of multisensory stimulation with BTS-Nirvana has on the neurodevelopment of a little girl affected by T8MS in association with agenesis of the corpus callosum. 

The methodology includes, in the psychodiagnostic phase, an assessment of the child’s development through direct observation and a survey of the different areas of neurodevelopment through the administration of the Griffith III Scale. In addition, direct observation of caregiver–child interaction and the collection of elements relating to the family context are carried out. The habilitative activity is articulated according to a conventional neuropsychomotor approach performed in association with non-conventional treatment with innovative high-tech tools with BTS-Nirvana. 

The BTS-Nirvana is a medical device based on virtual rehabilitation, specially designed for the rehabilitation of patients with neurological disorders who manifest motor and cognitive difficulties [24]. This system is based on optoelectronic infrared sensors, which allows the patients to interact in semi-immersive virtual reality scenarios, simply moving themself, improving their perceptive-cognitive abilities thanks to stimuli and audio-visual feedback and increasing motivation. The exercises can be customized in different ways, and therapists can increase levels of difficulty, adapting them to the abilities of the patient. The system is connected to a wall or floor projector, which reproduces interactive exercises, acting on motor and cognitive domains (attention, memory, spatial organization, balance and coordination). At the end of a work session, the tool allows you to export a database with the list of all the exercises that the patient has performed and the score he has obtained. The treatment consists of 50-min sessions once a week. Both scenarios with floor projections and scenarios with wall projections were used in order to work on multiple domains, i.e., identify or find objects, chase or move objects. 

Early recognition and monitoring of cognitive and behavioral difficulties have proven to be crucial in promoting the management and the definition of adequate therapeutic and rehabilitative strategies. The two-year follow-up showed important changes in neurodevelopment with positive effects on the child’s subsequent development. The therapeutic intervention specifically aimed at the child appears able to modify the evolutionary trajectory of a disorder, assuming a preventive value. 

Our study, in agreement with the literature [21,23], confirms the effects of early and innovative approach in the outcomes of children born without a corpus callosum by inducing remarkable brain plasticity.

## 2. Case Description

We reported a case of a 36-month-old female patient with T8MS, born to an assisted fertilization (FIVET). She was the first daughter of healthy, unrelated parents. On routine second trimester ultrasound examination, she showed ventriculomegaly and agenesis of the corpus callosum was confirmed. Subsequently, at encephalic MRI (30w of EG), she presented with evidence of mild ventriculomegaly on the left and marked on the right with a stroke of the lateral ventricles, agenesis of the corpus callosum and no other associated malformations. At amniocentesis, the finding of a female karyotype with mosaicism for chromosome trisomy 8: ”47, XX, +8[3]/46, XX [22] was performed de novo and was detected. Pregnancy complicated by hemorrhages was treated with bed rest and appropriate therapy, resulting in scheduled cesarean delivery at the 38th week +3 days. 

Anthropometric measures at birth: weight of 3500 g (75–90° pct), length 50 cm (75° pct) and occipital frontal circumference (OFC) of 33.5 cm (50° pct).

Physical examination at birth: mild dysmorphic features included scaphocephaly with splanchnocranium asymmetry with sn < dx and prominent forehead, deep-set eyes, large prominent ears with prominent antihelices, broad nose, high-arched palate, short frenum, abundant nuchal fold, clinodactyly IV toe of both feet and short metatarsals. The characteristic palmar and plantar creases were absent. Echocardiography showed patency of the foramen ovale/small atrial defect with mild left-to-right shunt. Mild dilation of the right sections was observed with normal biventricular global systolic function. Abdominal ultrasound, oculistic evaluation, audiological screening TEOAE and EEG did not show any congenital anomaly of the internal organs. The first evaluation of the development revealed cervico-axial hypotonia and four-limb hypertonia. No epileptic seizures are reported in history.

The child returned to the IRCCS Bonino Pulejo’s infantile neuropsychiatry service when she was 4 months old, having not yet acquired the normal stages of development. The child does not achieve neck holding, does not roll from supine to prone position and vice versa. Spontaneous movements in the four limbs are stereotypes, not very varied, due to the hypertonia that is most appreciated by the AAII. The little girl hooks her gaze on the object placed in front of her but does not hint at the movement of her upper limbs in an attempt to reach it. Verbal production is characterized by sounds and vocalizations that are sometimes organized in protoconversations. 

Ethics committee approval was not necessary; however, parents signed informed consent for scientific and data publication purposes.

## 3. Procedures

On the recommendation of the neuropsychiatrist, the girl was subjected to rehabilitation training thrice weekly, two conventional neuropsychomotor individual treatments and one complex instrumental one through the use of multisensory stimulation. Each session lasted 50 min. The study lasted 24 months. 

Motor and cognitive functions were assessed by the administration of the Griffiths III scales at the age of 6 (T0), 18 (T1), 24 (T2) and 30 months (T3). The early identification of symptoms that gradually occurred during development allowed us to apply rehabilitation programs based on previously standardized protocols and adapted according to the patient’s characteristics. Neuropsychomotor treatment was performed with the therapist of neuro- and psychomotricity. 

Conventional rehabilitation treatment included prevention of both internal (e.g., bronchitis) and external physical complications (e.g., muscle-tendon retraction, joint deformities) through assisted breathing exercises in the various decubitus, gentle passive mobilization and correct and diversified positioning, respectively.

Postural restraint was carried out through the use of cushions to reduce head extension and improve both the orientation of the head in the midline and the lengthening of the muscles of the posterior chain. Exercises to acquire control of the head and for correct alignment in the sitting posture were also combined with interventions on sensory, proprioceptive and vestibular afferents mediated through the body of the mother or therapist, in significant contexts, with the aim of both enriching the perceptual–motor patterns and the interaction and the mother–child relationship.

The objectives of the semi-immersive virtual reality treatment in an unconventional care setting (Nirvana) included in a first phase the stimulation of the child’s interest in the anterior space, proposing playful situations involving visual functions through pursuit of a person/object and the exploration of the environment using games in bright and contrasting colors, communication mediated by sounds games, vocalizations with pause codes and movement through body play and pleasure in being moved.

In particular, both scenarios with floor projections and scenarios with wall projections were used to work on multiple domains. For example, the “sprite” type exercises are very useful to recover visual-spatial skills and motor co-ordination by allowing the patient to explore the available environment with one or both upper limbs through movement and virtual grasp. Hunting exercise is indicated to improve attentional processes and eye–hand coordination. All exercises work on global motor skills, in particular to improve coordination and balance. 

On the recommendation of the neuropsychiatrist, the rehabilitation program was readjusted according to the patient’s characteristics (T1–T3), maintaining the three sessions of 50 min per week. Exercises were proposed to implement spatial cognition and attention, interpersonal and communication skills and perception and praxis through 3D computer-generated virtual scenarios, which ensured naturalistic and real-time body responses involving multiple sensory channels (tactile, auditory, visual and, sometimes, even gustatory and olfactory). The exercises were personalized in different ways, and the therapists have increased the levels of difficulty, adapting them to the patient’s abilities.

The exercises cover several virtual tasks: sprites, follow me, hunts, motion and virtual grasping. Sprite-type exercises allow the recovery of visual-spatial skills and spatial cognition, exploring scenarios with one or both upper limbs. Follow-me-type exercise stimulates the upper or lower limbs, also improving motor coordination. Hunt-type exercise allows you to improve attentional processes and eye–manual coordination. Motion-type exercise is aimed at controlling the limbs and trunk. As part of the therapeutic program, the involvement of parents was essential by suggesting activities to be included in everyday life to motivate the child to use her residue at any time of the day. 

The baby achieved neck control at 5 moths, sitting position at 9 months and independent standing position at 12 months old. She had slang language with few intelligible words.

At the time of consultation (T0), the neuropsychological assessment showed a disharmonious psychomotor profile characterized by high scores in the scales examining hearing–speech and personal-social aspects and low IQ in the performance scale, eye and hand coordination and the locomotion scale. 

**Figure 1:**
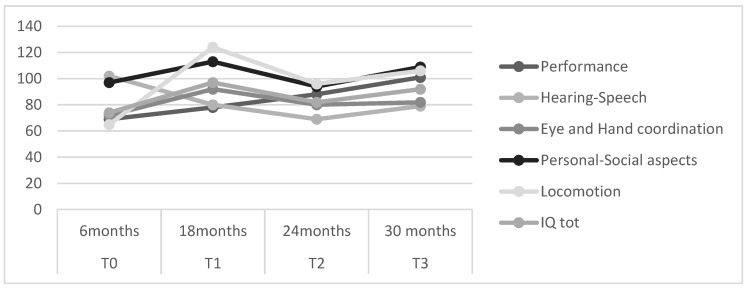
Line Plot. Representation of distribution of clinical assessment in each time.

Overall, the scores obtained after rehabilitative training (comparison T0–T3) revealed an improvement in performance on the individual tests (Table 1 and Figure 1). 

A Scale (Performance), B Scale (Hearing–Speech), C Scale (Eye and Hand coordination), D Scale (Personal-Social aspects), Scale E (Locomotion).

In particular, at T1 (18 months), the patient showed high improvements in scales examining locomotor aspect and oculo-manual coordination. Specifically, the child reached a sitting position at 9 months and standing at 12 months and started walking independently at the age of 14 months. A slight improvement was also recorded in the Performance Scale, which, although improved, remained less advanced than the expected average. The hearing–speech scale was below average. She had jargon speech with only few intelligible words. 

At the end of the first cycle of the rehabilitative training motor and cognitive functions were assessed at 24 and 30 months (T2 and T3). In particular, performance and locomotion scores showed an increasing trend at both times (T2 and T3). At T2, we found a slowdown in the hearing–speech and hand–eye coordination scales. Both scales improved in IQ scores at T3. The personal and social aspects score at all times showed a trend above the cut-off (>85). 

## 4. Discussion

Children and adolescents with T8MS look different and clinically distinctive because the chromosomal mosaicism, typical of this syndrome, determines variable phenotypes in affected subjects, from mild dysmorphism to severe structural abnormalities [25]. Multiple studies have focused attention on the simple description of the phenotype and structural anomalies including corpus callosum agenesis and renal malformations [26,27]. In clinical practice, subjects born with this rare disorder are usually treated to manage and contrast the complications that arise through traditional neurorehabilitation treatment, which includes speech therapy treatment, neuromotor treatment, occupational therapy and psychomotor treatment [20]. Recent studies on animal and human models have shown that rehabilitation approaches based on environmental enrichment exert profound effects on the CNS, particularly if applied in the early stages of development and if mediated by multisensory stimulation. Neuroplasticity mechanisms that allow functional reorganisation in the presence of neurodevelopmental disabilities are highlighted. These results are very promising in the field of neurorehabilitation [21,22]. The introduction of an innovative, non-invasive rehabilitation approach in pediatric rehabilitation plays an essential role in exploiting the effects of neuronal plasticity.

We describe a case of a girl with Warkany 2 syndrome (T8MS), associated with ventriculomegaly and agenesis of the corpus callosum, who underwent intense cognitive and motor rehabilitation training with a face-to-face treatment setting with the therapist associated with a medical device based on virtual rehabilitation (VR). In particular, Virtual Reality technology is an important new rehabilitation tool [22]. In this context, virtual reality (VR) technology is proposed as a new tool with a possible added value compared to traditional neurorehabilitation. 

Recent scientific studies have shown that the intensive and early stimulation of subjects born with agenesis of the corpus callosum induces structural neuroplastic responses, significantly reducing the impact that this malformation has on the child’s development, modifying the neurobehavioral outcomes that occur during development [20]. However, there are no data on cognitive and motor rehabilitation in patients with T8MS associated with ventriculomegaly and agenesis of the corpus callosum. 

In our study, the early identification of symptoms that gradually occurred during the development of the girl (T0, T1, T2) allowed us to apply innovative rehabilitation programs based on previously standardized protocols adapted according to the patient’s characteristics. 

We report our patient’s data after 24 months of treatment (T3). At T0, neuropsychological evaluation showed a disharmonious psychomotor profile, in line with literature data [5,20,28], characterized by impaired skills in performance, eye and hand coordination and locomotion. Rehabilitation treatment significantly promoted improvement in areas of development that were deficient. Indeed, at T1 (18 months), the patient showed high improvements in all development areas except performance and hearing–speech, which remain below average (see Table 1). The modification of the rehabilitation program based on the clinical characteristics that gradually occurred in the subsequent evaluations (T1 and T2) was influential and persistent (T3) in the Basics of Learning (A Scale), Personal-Social-Emotional (D Scale) and Grosso-motor (E Scale).

At T3, linguistic skills (B Scale) and oculo-motor coordination (C Scale) remained slightly below the average for age. Although the study is limited to a single case, we could support the idea that the stimulation of motor and cognitive skills with conventional treatment setting using face-to-face treatment with the therapist, associated with the innovative approach of rehabilitation with a complex instrumental using multisensory stimulation [21] with the BTS-Nirvana, could be promising for these patients. Our data support the idea that combining conventional treatment with Virtual Reality can be a promising tool for the rehabilitation of neurodevelopmental disorders.

VR technologies enable new rehabilitation opportunities in several diseases with cognitive and motor deficits, as virtual reality allows you to create protected environments and offers multisensory stimulation. So, further studies should be promoted, with a larger sample and a long-term follow-up, that allow evaluating how much functional gain is maintained. No side effects have been described in the literature for VR use.

## Figures and Tables

**Table 1:** Clinical assessment. Total score of neuropsychological and psychological assessment before and during rehabilitative training. T0 (baseline); T1 (12 months after T0); T2 (6 months after T1); T3 (12 months after T1).

Clinical Assessment Griffith Scale *	Case Study			
	T0	T1	T2	T3
Scale A	69	78	88	101
Scale B	102	80	69	79
Scale C	72	92	80	82
Scale D	97	113	94	109
Scale E	65	124	96	106
IQ tot	74	97	82	92

* Griffiths Mental Developmental Scales, from birth to 8 years of age.
